# A Short Report on the Lack of a Pyrogenic Response of Australian Genomic Group IV Isolates of *Coxiella burnetii* in Guinea Pigs

**DOI:** 10.3390/tropicalmed4010018

**Published:** 2019-01-25

**Authors:** Aminul Islam, John Stenos, Gemma Vincent, Stephen Graves

**Affiliations:** 1 Australian Rickettsial Reference Laboratory, University Hospital Geelong, Geelong, VIC 3220, Australia; johns@barwonhealth.org.au (J.S.); gvince@barwonhealth.org.au (G.V.); graves.rickettsia@gmail.com (S.G.); 2 New South Wales Health Pathology, Nepean Hospital, Penrith, NSW 2751, Australia

**Keywords:** *C. burnetii*, Q fever, Australia, pyrogenicity, guinea pigs

## Abstract

This small study reports on a non-pyrogenic response of five different Australian isolates of *Coxiella burnetii* (*C. burnetii*). They were all members of Genomic Group IV and obtained from three cases of acute human infection, one case of chronic human infection and one case of goat abortion. The guinea pigs infected with these isolates did not develop fever (temperature ≥ 40.0 °C), which is consistent with other members of this genomic group that were isolated from elsewhere in the world. In contrast, guinea pigs infected with the classical USA tick isolate, Nine Mile phase 1 (RSA 493) of Genomic Group I, experienced a four-day febrile period.

## 1. Introduction

Guinea pigs are an excellent small animal model of acute Q fever (infection with *Coxiella burnetii* or *C. burnetii*) in humans [1,2,3]. However, not all isolates of *C. burnetii* will cause fever (pyrogenicity) in guinea pigs. This feature of the bacterium appears to be related to the genomic group to which the isolate belongs, with group IV and VI known to be non-pyrogenic [4].

Recent Australian isolates of *C. burnetii* belong to a unique subset of genomic group IV; however, most were isolated from patients with acute Q fever, many of whom had presented with fever [5]. The question investigated in this small study was whether a selection of these Australian isolates were pyrogenic in guinea pigs.

## 2. Materials and Methods

### 2.1. Animal Ethics

This study was approved by the Australian Rickettsial Reference Laboratory Animal Care and Ethics Committee (ACEC/010). All experimental works were performed in a biosafety level 3 laboratory at the Department of Microbiology, John Hunter Hospital, Newcastle, New South Wales, Australia.

### 2.2. Coxiella Burnetii Isolates

Five Australian isolates of *C. burnetii* were selected for use in this study, with a range of molecular and epidemiological features (Table 1). All were members of genomic group IV, but represented three different genotypes (CbAu01, CbAu04 and CbAu06) according to a multi-locus variable number of tandem repeats (VNTR) analysis (MLVA). These genotypes were shown to be unique to Australia [5]. There were four human isolates that came from three cases of acute infection (AuQ01, AuQ10 and AuQ43) and one case of chronic infection (AuQ04). There was also one isolate from an aborting goat (AuQ57), which was associated with a number of human cases [6]. 

*C. burnetii* Nine Mile phase 1 (RSA493), originally obtained from a tick in the USA and belonging to Genomic Group I, was used as a positive control, as it was known to be pyrogenic in guinea pigs. Sterile cell culture medium (RPMI-1640) was used as a negative control. 

### 2.3. Culture and Quantification of C. burnetii in VERO Cell Line and Wild Mice

Vero cells were grown in 10ml RPMI (Gibco, Australia) supplemented with 10% new born calf serum (NBCS) (Gibco, Australia) and 1% l-glutamine (Gibco, Australia). The five *C. burnetii* isolates were inoculated into Vero cell monolayer and grown at 37 °C with 5% CO_2_ for 14 days. The infected monolayer was removed by scrapping and each preparation inoculated intraperitoneally into a single outbred mouse. This was done to ensure that all *C. burnetii* cells were in phase 1 (virulent) for the later guinea pig infection [7]. Each infected mouse was euthanized seven days later and its enlarged spleen removed aseptically. Each spleen was separately homogenized in 5 mL of Hank’s Balanced Salt Solution and the concentration of *C. burnetii* DNA measured by quantitative real-time PCR (qPCR) using an assay targeting the single-copy com1 gene [8]. Each spleen suspension was adjusted to contain between 10^6^ and 10^7^ *C. burnetii* per 0.2 mL. 

### 2.4. Experimental Guinea Pig Infection

Outbred breeds of adult male guinea pigs were used in this study (*n* = 24). One week prior to the start of the experiment, an IPTT300 temperature transponder (Biomedic Data Systems, Inc., Seaford, DE, USA) was implanted into the sub-cutaneous tissue on the flank of each guinea pig.

Each guinea pig was anaesthetized with a 0.2 mL intramuscular injection of 9.5 mL of ketamine (100 mg/mL) and 0.5 mL of xylazine (100 mg/mL). Each anaesthetized guinea pig had 0.2 mL of the infected mouse spleen suspension introduced slowly into its nostrils via a fine-bore plastic Pasteur pipette. The guinea pigs inhaled the liquid slowly and the *C. burnetii* presumably entered the animals’ lungs. 

### 2.5. Monitoring Guinea Pig Temperature with Probe

Guinea pig temperatures were recorded daily for 21 days using an IPTT-300 Smart Probe held over the location of the subcutaneous temperature transponder in the guinea pig. 

A temperature at or above 40 °C was defined as a fever and the guinea pig considered to be febrile. The experiment was terminated at day 21 post-infection by which time all guinea pig temperatures had returned to normal. 

## 3. Results

**Figure 1:**
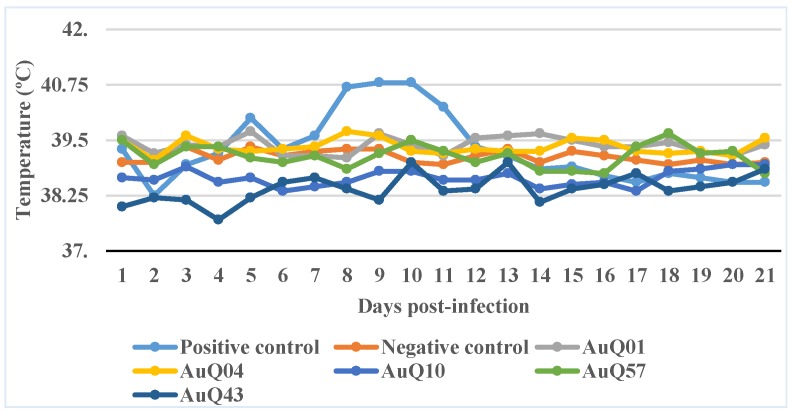
The temperature pattern of guinea pigs infected with five different Australian isolates of *C. burnetii*, with the positive (NM1) and negative (sterile RPMI 1640 media) controls over a period of 21 days post-infection. Febrile response (temperature ≥ 40.0 °C) in positive controls sustained from days 8–11 with an early onset at day 5. Throughout the experimental period, the five Australian isolates and negative control did not show febrile response and remained under 40.0 °C. The average value of temperature in each group is shown in the graph.

The temperature changes in the 24 guinea pigs (grouped according to the isolate of *C. burnetii* used to infect them) are shown in the Figure 1. The four guinea pigs given only Roswell Park Memorial Institute (RPMI) medium intranasally (negative controls) did not develop a fever at any stage. 

The four guinea pigs given *C. burnetii* Nine Mile phase 1 (positive control), developed fever (40.6 ± 0.3) from days 8–11 after infection (four-day duration). None of the five Australian *C. burnetii* isolates, inoculated into 16 guinea pigs, resulted in fever (38.6 ± 1.0). They were all non-pyrogenic. 

## 4. Discussion

The best small animal model for studying Q fever is the guinea pig, as it develops a fever of limited duration, similar to infected humans [3]. However, not all isolates of *C. burnetii* are pyrogenic in the guinea pig. Studies of non-Australian isolates show that those from genomic groups IV and VI are non-pyrogenic [4]. This study has now demonstrated the same phenomenon of non-pyrogenicity in five Australian isolates, which all belong to genomic group IV, albeit to a unique subgroup. The four human isolates used in this study caused fever in the human patients from whom the bacteria were obtained. However, they did not cause fever in the guinea pigs. While the pathological basis for this difference is not known, there is a practical significance to it. When using guinea pigs to test new vaccines for use against Q fever and using abrogation of fever as a clinical indicator of vaccine success, it is necessary to use a challenge isolate of *C. burnetii* that causes fever in the non-immunized guinea pigs. On the basis of this small study it will not be possible to use Australian isolates of *C. burnetii* for challenge in Q fever vaccine studies, if abrogation of fever is used as a clinical marker of vaccine protection. It appears that the Nine Mile phase 1 isolate of *C. burnetii* is required for this purpose. 

## 5. Conclusions

Australian isolates of *C. burnetii* do not cause fever in experimentally infected guinea pigs, confirming what has been shown in other genomic group IV isolates elsewhere in the world.

## Figures and Tables

**Table 1:** Brief molecular and epidemiological features of the five Australian isolates of *Coxiella burnetii* (*C. burnetii*) used in the pyrogenicity study.

*C. burnetii* Isolates	MLVA * Genotype	Epidemiological Features					
		Type of Q Fever	Year	Location	Animal Contact	Symptoms	Source of Isolate
AuQ01 (Human)	CbAu01	Acute	2005	Armidale, Northern NSW	Goat	Fever, Jaundice	Blood
AuQ04 (Human)	CbAu04	Chronic	2007	Swan Hill, Northern VIC	Unknown	Fever, Endocarditis/Aortic valve incompetence	Surgically removed tissue
AuQ10 (Human)	CbAu06	Acute	2011	Coffs Harbour, Northern NSW	Unknown	Fever, Haemophagocytic	Blood
						Syndrome	
AuQ43 (Human)	CbAu01	Acute	2012	Mt Louisa, Northern QLD	Unknown	Fever	Blood
AuQ57 (Animal)	CbAu01	Goat coxiello-sis	2012	Meredith, Central VIC	Goat	Abortion	Aborted foetus

* MLVA-multiple locus variable number of tandem repeats analysis.

## References

[1] Scott G.H., Burger G.T., Kishimoto R.A. (1978). Experimental *Coxiella burnetii* infection of guinea pigs and mice. Lab. Anim. Sci..

[2] Russell-Lodrigue K.E., Zhang G.Q., McMurray D.N., Samuel J.E. (2006). Clinical and pathologic changes in a guinea pig aerosol challenge model of acute Q fever. Infect. Immun..

[3] Bewley K.R. (2013). Animal models of Q fever (*Coxiella burnetii*). Comp. Med..

[4] Russell-Lodrigue K.E., Andoh M., Poels W.J., Shive H.R., Weeks B.R., Zhang G.Q., Tersteeg C., Masegi T., Hotta A., Yamaguchi Y. (2009). *Coxiella burnetii* isolates cause genogroup-specific virulence in mice and guinea pig models of acute Q fever. Infect. Immun..

[5] Vincent G., Stenos J., Latham J., Fenwick S., Graves S. (2016). Novel genotypes of *Coxiella burnetii* identified in isolates from Australian Q fever patients. Int. J Med. Micro..

[6] Bond K.A., Vincent G., Wilks C.R., Franklin L., Sutton B., Stenos J., Cowan R., Lim K., Athan E., Harris O. (2016). One health approach to controlling a Q fever outbreak on an Australian goat farm. Epidemiol. Infect..

[7] Ormsbee R.A., Bell E.J., Lackman D.B., Tallent G. (1964). The influence of phase on the protective potency of Q fever vaccine. J. Immunol..

[8] Lockhart M.G., Graves S.R., Banazis M.J., Fenwick S.G., Stenos J. (2011). A comparison of methods for extracting DNA from *Coxiella burnetii* as measured by a duplex qPCR assay. Lett. Appl. Microbiol..

